# Diagnostic Accuracy of Plasma p-tau217 for Detecting Pathological Cerebrospinal Fluid Changes in Cognitively Unimpaired Subjects Using the Lumipulse Platform

**DOI:** 10.14283/jpad.2024.152

**Published:** 2024-08-07

**Authors:** Francisco Martínez-Dubarbie, A. Guerra-Ruiz, S. López-García, C. Lage, M. Fernández-Matarrubia, J. Infante, A. Pozueta-Cantudo, M. García-Martínez, A. Corrales-Pardo, M. Bravo, M. López-Hoyos, J. Irure-Ventura, E. Valeriano-Lorenzo, M. T. García-Unzueta, P. Sánchez-Juan, E. Rodríguez-Rodríguez

**Affiliations:** 1 Neurology Service, Marqués de Valdecilla University Hospital, Avda. de Valdecilla 25, 39008 Santander, Cantabria Spain; 2 Institute for Research Marqués de Valdecilla (IDIVAL), 39011 Santander, Cantabria Spain; 3 CIBERNED, Network Center for Biomedical Research in Neurodegenerative Diseases, National Institute of Health Carlos III, 28220 Madrid, Spain; 4 Biochemistry and Clinical Analysis Department, Marqués de Valdecilla University Hospital, 39008 Santander, Cantabria Spain; 5 Atlantic Fellow for Equity in Brain health, Global Brain Health Institute, University of California, San Francisco, 94158 USA; 6 Medicine and Psychiatry department, University of Cantabria, 39011 Santander, Spain; 7 Universidad Europea del Atlántico, 39011 Santander, Spain; 8 Immunology Department, Marqués de Valdecilla University Hospital, 39008 Santander, Spain; 9 Molecular Biology Department, University of Cantabria, 39011 Santander, Spain; 10 Alzheimer’s Centre Reina Sofia-CIEN Foundation-ISCIII, 28031 Madrid, Spain

**Keywords:** Alzheimer’s disease, plasma p-tau217, lumipulse platform, cognitively unimpaired individuals, preclinical stages

## Abstract

**Background:**

Plasma biomarkers of Alzheimer’s disease (AD), especially p-tau217, are promising tools to identify subjects with amyloid deposition in the brain, determined either by cerebrospinal fluid (CSF) or positron emission tomography. However, it is essential to measure them in an accurate and fully automated way in order to apply them in clinical practice.

**Objectives:**

To evaluate the diagnostic performance of the fully-automated Lumipulse plasma p-tau217 assay in preclinical AD.

**Design:**

Cross-sectional analyses from a prospective cohort.

**Setting:**

A population-based study.

**Participants:**

Volunteers over 55 years without cognitive impairment or contraindications for complementary tests.

**Measurements:**

Plasma p-tau217 was measured with the fully-automated Lumipulse assay, as well as CSF Aβ40, Aβ42, p-taul81, and t-tau levels. We correlated plasma p-tau217 with CSF Aβ40, Aβ42 and p-tau181, and assessed the differences in plasma p-tau217 according to CSF amyloid status (A−/+), AD status (AD+ being those subjects A+T+ and AD- the rest) and ATN group. We performed ROC curves and measured the areas under the curve (AUC) using CSF amyloid as result, and both p-tau217 and ApoE4 status as predictor.

**Results:**

We screened 209 cognitively unimpaired volunteers with a mean age 64 years (60–69) and 30.2% of ApoE4 carriers. Plasma p-tau217 correlated significantly with CSF Aβ42/Aβ40 (Rho=−0.51; p-value<0.001) and p-tau181 (r=0.59; p-value<0.001). Its levels were significantly higher in A+ subjects (0.26 pg/ml) compared with A- (0.12 pg/ml; p-value<0.001); and along ATN groups. It predicts CSF amyloid pathology with an AUC of 0.85.

**Conclusions:**

Plasma p-tau217 measured using the Lumipulse platform shows promise as an accurate biomarker of preclinical AD pathology.

**Electronic Supplementary Material:**

Supplementary material is available in the online version of this article at 10.14283/jpad.2024.152.

## Introduction

Alzheimer’s disease (AD) has undergone a revolution in recent years with the approval of the first disease-modifying drugs by the U.S. Food and Drug Administration and the development of plasma biomarkers. AD is histologically defined by the brain deposition of beta-amyloid (Aβ) and phosphorylated tau (p-tau) proteins in extracellular plaques and intracellular neurofibrillary tangles, respectively (1, 2). These brain changes can be identified in vivo by imaging tests such as amyloid positron emission tomography (PET) and tau PET, and also by cerebrospinal fluid (CSF) analysis (2–4).

These imaging and CSF diagnostic tests represented an important breakthrough in the diagnosis of the disease, since they have allowed a better understanding of its pathophysiology, classifying the disease from a biological point of view, and identifying patients with pathological changes before they develop the first symptoms (2). In this regard, studies in genetic forms of AD have shown that brain changes can occur decades before clinical onset (5). However, conventional diagnostic techniques are expensive, invasive, and not widely available.

Recently, it has become technically possible to analyze biomarker levels in plasma. They reflect the different stages of the amyloid cascade (Aβ42/Aβ40 ratio, p-tau181, p-tau217, and p-tau231), axonal damage (Neurofilament Light Chain (NfL)) and astrocyte reactivity (Glial Fibrillary Acidic Protein) (6–12). Each biomarker provides complementary information about the disease, and it is expected that their combined use will allow early diagnosis, treatment response monitoring and differential diagnosis in an inexpensive, minimally invasive, and accessible way (13).

Among these biomarkers, one of the most promising is p-tau217, which has been shown to alter very early in plasma and reflects with great precision amyloid deposition at the brain level measured by CSF and PET (8). In research including anatomopathological examinations, this biomarker has been shown to be associated with both amyloid and tau pathology (14, 15). It is also an AD-specific biomarker that shows greater fold-change in AD patients than other biomarkers such as p-tau181, NfL, and the Aβ42/Aβ40 ratio, so is less affected by analytical fluctuations (15, 16). Moreover, its levels seem to increase progressively along the AD continuum, relating to progressive brain atrophy and cognitive impairment (8, 17).

Plasma p-tau217 has been also evaluated and compared with p-tau181 and p-tau231 by different immunoassays in a memory clinic population and has shown strong results for detecting CSF Alzheimer’s pathology with performances similar to those of CSF p-tau217 (18). In addition, it has recently been shown that plasma p-tau217 has a similar performance to established CSF biomarkers for detecting amyloid pathology and a superior performance for tau pathology in subjects with mild cognitive impairment (19). Also, already available commercially plasma p-tau217 immunoassays can identify both CSF amyloid and tau pathology with great accuracy and showed longitudinal changes through the AD continuum even in preclinical stages (20).

Although plasma biomarkers are only available in the research setting, it is expected that they can be incorporated into clinical practice as information on their various characteristics becomes available (13). In an attempt to provide practical data, some authors have suggested a two-step workflow. In subjects with mild cognitive impairment, they have used plasma biomarkers first, and only performed LP in those subjects with indeterminate results, thus suggesting that 61.2–85.9% of the LPs performed in daily practice can be avoided (21). These strategies may be useful in cognitively healthy populations, which are particularly challenging.

In addition, assessing the results of devices already available in many hospitals is essential to facilitate access to plasma biomarkers and to use them on a large scale. Using fully-automated platforms also adds robustness to the data as it minimizes analytical variations and makes the findings more reproducible. Our aim was to assess the ability of plasma p-tau217 to identify AD pathological CSF changes of cognitively unimpaired (CU) subjects using Fujirebio’s fully automated and widely available Lumipulse platform. For this purpose, we have analyzed the performance of p-tau217 and also compared it with both the plasma Aβ42/Aβ40 ratio and p-tau181, biomarkers that have already been explored and have shown very good results under these conditions (22).

## Methods

### Study participants

The study was performed with volunteers of the ‘Valdecilla Cohort for the study of memory and brain aging’ from the Memory Unit of the Marqués de Valdecilla University Hospital (Santander, Spain), and it was approved by the ethics committee of this hospital (Internal code: 2018.111). The project has been described in previous articles (23). In brief, it is a cohort designed to longitudinally study the preclinical phases of AD. It is composed of Caucasian CU subjects over 55 years of age who have signed an informed consent for the collection and storage of biological samples. Exclusion criteria are the presence of any degree of cognitive impairment, defined by a Clinical Dementia Rating (CDR) (24) >0; major psychiatric or systemic disease; sensory impairment that hinders the performance of cognitive tests; any contraindication for performing the complementary tests (e.g. claustrophobia or anticoagulation).

At baseline, all participants undergo a lumbar puncture (LP) to measure Aβ42, Aβ40, p-tau181 and total tau (t-tau), and blood draw. In the first assessment, a cranial magnetic resonance imaging (MRI), fluorodeoxyglucose PET, and a comprehensive neuropsychological (NPS) study are also performed. Annual follow-ups consist of a new blood draw and NPS evaluation.

Regarding the detailed selection process of the participants, 328 subjects responded to an open call in the media of our community. Of those, 62 participants are pending a first evaluation. Among the remaining 266, 26 were excluded because of unavailability (due to schedules or changes in the place of residence); 13 were excluded because they were unable to undergo MRI or LP; 8 because they presented systemic or psychiatric pathology; 3 due to imaging test findings (space-occupying lesions or major stroke); and 7 were excluded because they had a score >0 on the CDR scale. Finally, 209 subjects were studied for CSF Aβ42, Aβ40 and p-tau181 and for plasma p-tau217 levels. A graphical flowchart of the selection process can be seen in Supplementary Figure 1.

### Cognitive assessment

The neuropsychological assessment consists of a battery with sensitive tests that evaluate all cognitive domains. The details have been described in a previous work (25). The Mini-Mental State Examination (MMSE) (26) is used for a global cognitive assessment and the global CDR score is used to establish the degree of dementia based on functionality and cognition.

### ApoE status determination

We studied the apolipoprotein E (ApoE) genotype using TaqMan single nucleotide polymorphism genotyping assay (Applied Biosystems, Foster City, CA, United States). Subjects carrying ≥1 copy of the ε4 allele were considered ε4+ and the rest fell into the ε4- group.

### Sample collection and pre-analysis

Our institution is part of the Alzheimer’s Association Quality Control program, so we follow the international recommendations for sample collection and storage (27, 28). CSF and plasma samples are taken the same day, between 9–10 AM, less than 30 minutes apart and with the subjects fasting. The LPs are performed using a standard 22G needle, between the L3-L5 spaces, and lateral decubitus. The CSF is collected into polypropylene tubes of 15 mL and centrifuged at room temperature (2000 g for 10 minutes). The resultant is aliquoted in 500 *µ*l volumes into 1 mL tubes and frozen at −80 °C until analysis in the immunology laboratory of the Marqués de Valdecilla University Hospital.

Plasma samples are obtained following the standardized operating procedure detailed in previous works (29). The blood is stored in EDTA tubes of 10 mL and stored cold until processing within three hours. The samples are centrifuged (10 minutes at 1800 g). The supernatant is stored in 500 *µ*l volumes in polypropylene tubes and frozen at −80 °C until analyzed in the biochemistry laboratory of the Marqués de Valdecilla University Hospital.

### CSF and plasma biomarkers

CSF Aβ40, Aβ42, p-tau181 and t-tau levels were measured using the automated immunoassay analyzer Lumipulse G600 II (30) (Fujirebio Diagnostics, Malvern, PA, United States). The following kits were used: Lumipulse G β-Amyloid 1–40, Lumipulse G β-Amyloid 1–42, Lumipulse G p-tau181 and Lumipulse G t-tau.

To establish the CSF cut-off points we used an unbiased Gaussian mixture modelling based on a cohort of 578 subjects comprising both CU and cognitively impaired subjects (31). Analytical features, including lower limit of detection, inter- and intra- assay variations for Aβ40, Aβ42, p-tau181 and t-tau can be found elsewhere (22). Based on this model, subjects were divided according to the ATN classification (32). We considered Aβ-positive (A+) when CSF Aβ42/40 ratio <0.067, taupositive (T+) when CSF p-tau181 >55.0 pg/mL, and neurodegeneration-positive (N+) when CSF t-tau >389 pg/mL. When Nx is indicated, it means that both N+ and N− subjects are included. For the analyses, in addition to splitting subjects into A+ and A−, we segregated subjects into those with biologically defined Alzheimer pathology (A+T+) as AD+ and the remaining ones as AD−.

Plasma Aβ40, Aβ42, p-tau217, and p-tau181 levels were also measured using Fujirebio’s Lumipulse G600II. The following kits were used: Lumipulse G β-Amyloid 1–40 Plasma (lot T4B3033), Lumipulse G β-Amyloid 1–42 Plasma (lot T6B3074), Lumipulse G pTau 217 Plasma (lot D4C4097) and Lumipulse G pTau 181 Plasma (lot T9B3084). For Aβ40 and Aβ42, analytical sensitivity were 0.44 and 0.37 pg/ml, respectively; and intraassay variations were <3.1 and <3.8. Inter-run variability was <3.6 for Aβ40 and <4.7 for Aβ42. Analytical sensitivity for p-tau217 and p-tau181 were 0.030 and 0.052 pg/mL respectively. Intra- and inter- assay variations were <2.3% and <3.9% for p-tau181 and 3.2% and 5.7% for p-tau217. All the samples were above lower limit of detection.

### Physiological variables and comorbidities

To assess the influence of different variables on plasma biomarkers levels, we took into account renal filtration rate and body mass index and also the previous diagnosis of arterial hypertension and diabetes mellitus. As continuous variables, we have considered the estimated glomerular filtration rate (eGFR) and the body mass index (BMI). The eGFR was obtained through the Chronic Kidney Disease Epidemiology Collaboration formula (33) and expressed in mL/min/1.73m^2^; the BMI was calculated with the height (m) and weight (kg) collected at the initial assessment and expressed in kg/m^2^. Participants were also dichotomized according to their medical records into those who had previous history of hypertension (HT) and diabetes mellitus (DM) and those who had not.

### Statistical analysis

Shapiro-Wilk test was used to assess the distribution of the variables. According to these results, they have been described by mean and standard deviation (SD) or median and interquartile range (IQR), as applicable. Both plasma and CSF biomarkers were log10-transformed for analysing them with parametric tests.

We used Pearson’s correlation coefficient to correlate CSF Aβ40, Aβ42, and p-tau181 values with plasma p-tau217 in the overall sample and stratifying by A+ and A− subjects. Since CSF Aβ42/40 ratio and plasma p-tau217 have a non-linear relationship, we used the Spearman’s correlation coefficient.

Next, ANCOVA was used to study the potential of plasma p-tau217 to detect changes in CSF. For this purpose, we chose plasma p-tau217 as dependent variable, amyloid (A+ or A−) and tau (T+ or T−) status as independent variables, and age and sex as covariates. We also stratified participants according to ATN group. In this step, we considered the A−T−N−, A+T−N−, A−T+Nx, and A+T+Nx clusters. We assessed the overall differences between groups with ANOVA. If differences were found, a post hoc analysis was further performed with Tukey’s test to analyze differences between groups. The effect size of these differences was determined using Cohen’s d.

To test individual plasma biomarker diagnostic performances, we have developed univariate logistic regression models taking A−/+ and AD−/+ as response and the different plasma biomarkers as predictors. We have built receiver operating characteristic (ROC) curves and compared their area under the curve (AUC) with the DeLong test. Subsequently, we selected p-tau217 to perform a multivariate model. Thus, we have chosen plasma p-tau217 levels, age and ApoE4 status as predictors and, with this full model we have performed bootstrapping (n=1000 bootstrap samples) to make a backward variable selection using a p-value=0.157 for the likelihood ratio test (34).

To test the two-step workflow suggested in other articles (21), we selected logistic regression model with the best results to predict CSF amyloid pathology. By bootstrapping we selected different cut-off points for each model for sensitivities and specificities of 90, 95 and 97.5% and stratified subjects into those at high risk of pathology, low risk and indeterminate risk. With these data we also calculated the false positive and false negative rate.

The influence of eGFR and BMI on plasma p-tau217 was assessed through a multiple linear regression model adjusted by age and sex, in the overall sample and stratifying by A+/− group. We have also used ANCOVA to analyze differences in plasma p-tau217 as a function of HT and DM, adjusting by age and sex.

Those results with a p-value<0.05 were considered statistically significant. Missing values have been handled by omission. All statistical analyses were performed with R studio software version 4.2.22 (R Foundation for Statistical Computing, Vienna, Austria).

## Results

### Sample description

Our sample was composed of 209 participants, 137 females (65.5%) and 72 males (34.5%). The median age was 64 years (IQR 60–69). 62 participants (30.2%) were carriers of at least one ApoE4 allele and all subjects were CU, with a median MMSE of 29 (IQR 28–30). Both CSF and plasma biomarker values and number of subjects in each ATN group are shown in Table 1.

**Table 1:** Sample characteristics

**Characteristic**	**n=209**
Females, n. (%)	137 (65.5%)
Age, median (IQR)	64 (60–69)
ApoE ε4 carrier, n. (%)	62 (30.2%)
MMSE (0–30), median (IQR)	29 (28–30)
CSF Biomarkers	
Aβ40, mean (SD), pg/mL	10889.4 (3207.9)
Aβ42, median (IQR), pg/mL	820.0 (578–1036)
Ratio Aβ42/40, median (IQR)	0.084 (0.064–0.093)
T-tau, median (IQR), pg/mL	318 (243–400)
P-tau181, median (IQR), pg/mL	36.7 (30.4–54.4)
Plasma Biomarkers	
P-tau217, median (IQR), pg/mL	0.1 (0.09–0.16)
P-tau181, median (IQR), pg/mL	1.1 (0.9–1.4)
Ratio Aβ42/Aβ40, median (IQR)	0.082 (0.074–0.088)
ATN group, n. (%)	
A−T−N−	140 (66.9%)
A+T−N−	19 (9.1%)
A−T+N−	11 (5.3%)
A+T+Nx	39 (18.6%)
Physiological variables and comorbidities	
eGFR, median (IQR), mL/min/1.73m^2^	93.7 (86.7–98.3)
Body mass index, median (IQR), kg/m^2^	26.3 (23.9–29.0)
Hypertension (% yes)	37.9
Diabetes mellitus (% yes)	8.4

Abbreviations: n, number of subjects. IQR, interquartile range. SD, standard deviation. MMSE, mini-mental state examination. CSF, cerebrospinal fluid. Aβ, amyloid beta. T-tau, total tau. P-tau, phosphorylated tau. A, amyloid. T, tau. N, neurodegeneration. eGFR, estimated glomerular filtration rate.

### Influence of physiological variables and comorbidities on plasma biomarkers

**Figure 1:**
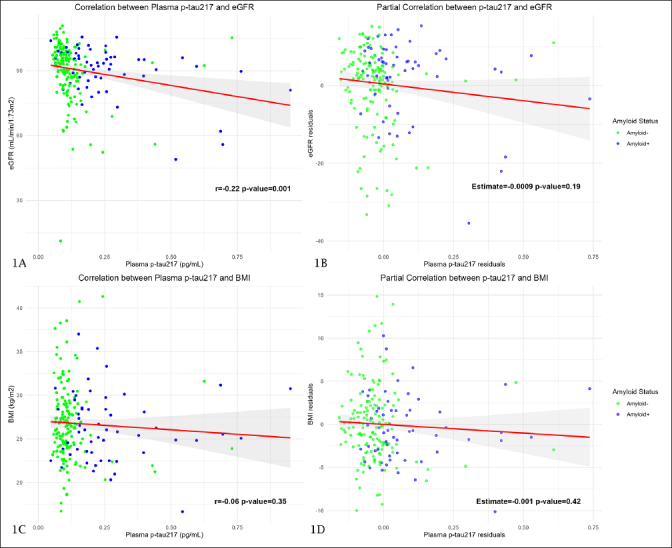
Correlation between plasma p-tau217 and physiological variables Figure 1A and 1C show Pearson’s correlation coefficient between plasma p-tau217 and eGFR and BMI, respectively. The Y axis corresponds to eGFR and BMI values and the X axis to plasma p-tau217 levels. The dots represent a pair of values of both variables for each observation. The green ones are those corresponding to amyloid-negative subjects and the blue ones represent the amyloid-positive subjects. The red line is the regression line, and the shaded area represents the 95% confidence interval. Figure 1B and 1D are scatterplots representing the relationship between plasma p-tau217 and eGFR and BMI, respectively, after adjusting for age. In these plots, the X axis represents the residuals of the linear regression of plasma p-tau217 on age (variability of p-tau217 that cannot be explained by age), and the Y axis shows the residuals of eGFR and BMI. Abbreviations: P-tau, phosphorylated tau. eGFR, estimated glomerular filtration rate. BMI, body mass index.

The correlation between eGFR and plasma p-tau217 was significant in the whole sample (r=−0.22; p-value=0.001) (Figure 1A). However, when we considered the influence of age, eGFR did not show a significant influence on plasma p-tau217 values in the overall sample (Estimate=−0.0009 pg/mL; p-value=0.19) (Figure 1B) and neither did it in A− (Estimate=−0.0007 pg/mL; p-value=0.21) nor in A+ (Estimate=−0.004 pg/mL; p-value=0.06) subjects. The correlation between BMI and plasma p-tau217 was not significant in the whole sample (r=−0.06; p-value=0.35) (Figure 1C) and the same happened when we considered the influence of age. BMI did not show a significant influence on plasma p-tau217 neither in the overall sample (Estimate=−0.001 pg/mL; p-value=0.42) (Figure 1D) nor in A− (Estimate=−0.0009 pg/mL; p-value=0.57) or A+ (Estimate=-0.0009 pg/mL; p-value=0.87) subjects.

We found no significant differences in p-tau217 values in subjects with and without HT (p-value=0.09) and in subjects with and without DM (p-value=0.85), so we did not perform a post-hoc study.

### Correlation between plasma p-tau217 and CSF biomarkers

**Figure 2:**
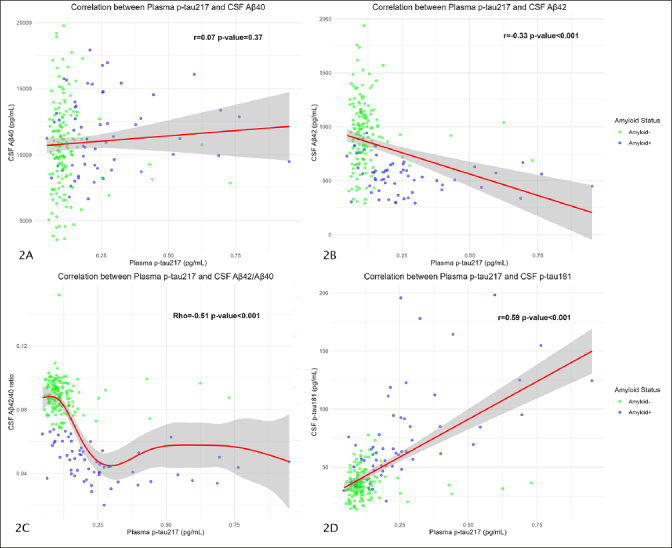
Correlations between plasma p-tau217 and CSF biomarkers The plots show Pearson’s correlation coefficient between plasma p-tau217 and CSF Aβ40 (Figure 2A), CSF Aβ42 (Figure 2B), and CSF p-tau181 (Figure 2D). Figure 2C shows the Spearman’s correlation between CSF Aβ42/Aβ40 ratio and plasma p-tau217. The Y axis corresponds to CSF values and the X axis to plasma values (all, except the amyloid ratio, expressed in pg/mL). The dots represent a pair of values of both variables for each observation. The green ones are those corresponding to amyloid-negative subjects and the blue ones represent the amyloid-positive subjects. The red line is the regression line, and the shaded area represents the 95% confidence interval. Abbreviations: CSF, cerebrospinal fluid. Aβ, amyloid β. P-tau, phosphorylated tau.

We correlated plasma p-tau217 values with the different CSF biomarkers. In the overall sample, plasma p-tau217 was not significantly correlated with CSF Aβ40 (r=0.07; p-value=0.37) (Figure 2A). These results were also not statistically significant in the A- group (r=0.05; p-value=0.51) nor in the A+ group (r=0.04; p-value=0.75).

The correlation between plasma p-tau217 and CSF Aβ42 was statistically significant in the overall sample (r=−0.33; p-value<0.001) (Figure 2B), but these results were not significant when dividing into A− (r=−0.07; p-value=0.36) and A+ (r=−0.24; p-value=0.06) groups.

We found a significant correlation between plasma p-tau217 and CSF Aβ42/Aβ40 ratio in the overall sample (Rho=−0.51; p-value<0.001) (Figure 2C) and both the A+ (Rho=−0.55; p-value<0.001), and A- groups (Rho=−0.16; p-value=0.04).

Finally, the correlation with CSF p-tau181 was significant in the overall sample (r=0.59; p-value<0.001) (Figure 2D) and in the A+ subjects (r=0.61; p-value<0.001), but not in the A− group (r=0.02; p-value=0.78). The remaining correlations between plasma p-tau181 an Aβ42/Aβ40 ratio are represented in Supplementary Table 1.

### Differences in plasma p-tau217 according to amyloid (A+) and AD (A+T+) status

**Figure 3:**
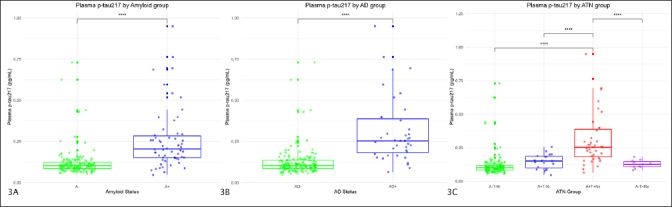
Differences in plasma p-tau217 according to amyloid status, AD status and ATN group The figure shows box and whiskers plots of plasma p-tau217 by groups. The X axis represents the different groups according to the CSF (figure 3A corresponds to Amyloid status, figure 3B to AD status, and figure 3C to ATN groups). Y axis corresponds to plasma p-tau217 concentrations expressed in pg/ml. The boxes show the interquartile range (the upper boundary is the Q3, and the lower boundary is the Q1). The line inside the box corresponds to the median of the sample and the whiskers represent the maximum (upper) and minimum (lower) values. The dots indicate individual values. Significant differences are indicated with a horizontal line and three asterisks between the boxes. Abbreviations: AD, Alzheimer’s Disease. A, amyloid. P-tau, phosphorylated tau. T, tau. N, neurodegeneration.

We also analyzed the differences in mean plasma p-tau217 values between subjects considered A+ and A− and both AD− and AD+ adjusting for age and sex. In the linear model, the influence of the amyloid group on plasma p-tau217 values was significant (Estimate=0.13; p-value<0.001) so a group analysis was performed. It showed that mean p-tau217 values were higher in A+ subjects (0.26 pg/ml) than in A− (0.12 pg/ml; p-value<0.001; Cohen’s d=1.18 with a 95% confidence interval (CI) 0.8–1.5) (Figure 3A).

Regarding plasma p-tau217 levels by AD group, in the model adjusted for age and sex, the AD group showed a significant influence (Estimate=0.18; p-value<0.001). In the subsequent study by groups, it was seen that the mean p-tau217 levels in AD+ subjects were significantly higher (0.32 pg/ml) than in AD− subjects (0.12 pg/ml; p-value<0.001; Cohen’s d=1.74 with a 95%CI 1.3–2.1) (Figure 3B).

### Differences in plasma p-tau217 according to ATTN group

Participants considered as A−T−N− showed a mean plasma p-tau217 concentration of 0.12 pg/mL (SD=0.09). A+T−N− subjects had a mean value of 0.15 pg/mL (SD=0.05); the A−T+N- had a mean value of 0.13 pg/mL (SD=0.003), and those classified as A+T+Nx had a mean concentration of plasma p-tau217 of 0.32 pg/ml (SD=0.19)

We found statistically significant differences in the mean plasma p-tau217 values between different ATN groups we found significant differences (p-value<0.001), so we performed a post-hoc study (Figure 3C). The difference between A−T−N− and A+T−N− subjects was not significant (0.028 pg/ml; p-value=0.74), neither it was between A−T−N− and A−T+Nx subjects (0.007 pg/ml; p-value=0.99). However, the difference was significant between A−T−N− and A+T+Nx subjects (0.2 pg/ml; p-value<0.001; Cohen’s d=1.7 with a 95%CI 1.3–2.1).

The difference between A+T−N− and A+T+Nx subjects was significant (0.17 pg/ml; p-value<0.001; Cohen’s d=1.0 with a 95%CI 0.4–1.6), but we found no differences between A+T−N− and A−T+Nx individuals (0.02 pg/ml; p-value=0.96). We found significant differences between A+T+Nx and A−T+Nx subjects (0.19 pg/ml; p-value<0.001; Cohen’s d=1.1 with a 95%CI 0.3–1.8).

### Comparison of different plasma markers to detect CSF pathology

Next, we assessed the performance of different plasma markers individually to detect CSF pathology. First, in an exploratory manner, we have analyzed whether a model that includes plasma p-tau217 along with age and sex provides diagnostic benefit with respect to age and sex alone. In this sense, for predicting amyloid status, the addition of p-tau217 showed a significant AUC improvement (0.83 vs. 0.68; p-value<0.001). When we assessed the ability of plasma p-tau217 alone to differentiate between A− and A+ subjects, the AUC was 0.85 (95%CI 0.78–0.92) with an optimal cut-off point of 0.25 (corresponding to 0.095 pg/mL; specificity=0.88, sensitivity=0.79).

The AUC of plasma p-tau181 by itself to detect amyloid pathology in CSF was 0.75 (95%CI 0.67–0.82) with an optimal cut-off point of 0.27 (corresponding to 1.23 pg/mL; specificity=0.76, sensitivity=0.65). When we compared it directly with the AUC of p-tau217 (0.85) we found the latter to be significantly higher (p-value=0.001).

When we analyzed the ability of plasma amyloid ratio to discriminate A− from A+ subjects, the AUC was 0.9 (95%CI 0.86–0.95) with an optimal cut-off point at 0.23 (equivalent to a ratio of 0.087; specificity=0.78 and sensitivity=0.86). However, when comparing the AUC with that of p-tau217, it was not significantly higher (p-value=0.14).

Since plasma p-tau217 was the marker which performed best, it is the one we have used in the following models.

### Performance of plasma p-tau217 for detecting CSF amyloid pathology

After testing the ability of p-tau217 in plasma alone to detect CSF amyloid pathology, we also included age and ApoE4 status as a full model and performed a backward variable selection through bootstrapping (Supplementary Table 2). The model providing best results for detecting amyloid pathology was the one including plasma p-tau217 levels together with ApoE4 status, with an AUC of 0.88 (95% CI= 0.83–0.93) and an optimal cutoff point of 0.13 (specificity=0.72; sensitivity=0.93).

### Use of the two-step model in our population

To apply the two-step workflow for detecting CSF amyloid pathology, we have selected the model with the best results (the one that takes both p-tau217 and ApoE4 status as independent variables). We have adjusted the sensitivities and specificities to 90%, 95% and 97.5% respectively, and analyzed how many subjects were classified above, below and between the thresholds.

**Figure 4:**
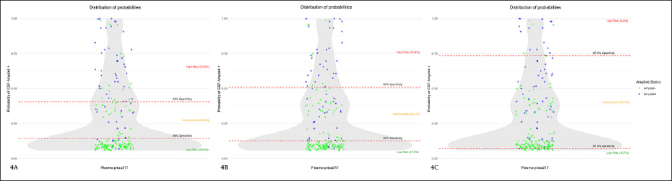
Two-step workflow application in our sample The results are shown in violin plots. The Y axis is a zero-to-one scale which represents the probability of being considered amyloid positive according to a logistic regression model in which amyloid status was taken as the response and plasma p-tau217 values and ApoE4 status as the predictive variables. The grey shaded area represents the concentration of observations at each probability. The dots are the individual values of plasma p-tau217. Those subjects considered as amyloid-positive according to CSF are blue dots and the amyloid-negative are the green ones. The red lines represent the cut-off point of the different specificities and sensitivities (90% in Figure 4A, 95% in Figure 4B, and 97.5% in Figure 4C). Values above the specificity line are considered «high risk», those below the sensitivity line are «low risk» and values between the lines are subjects classified as indeterminate.

Placing the sensitivity and specificity at 90% for detecting CSF amyloid pathology, 53 subjects (25.3%) were classified as high risk, 39 (18.9%) as low risk, and 117 (55.8%) as intermediate risk. Of subjects classified as high risk, 15 (7.2% of the total sample) were A− and therefore false positives (FP). Of those classified as low risk, 6 (2.9% of the total) were A+ and therefore false negatives (FN) (Figure 4A). The positive predictive value (PPV) and negative predictive value (NPV) were 0.68 and 0.95, respectively. The global accuracy in this case was 0.77.

When placing sensitivity and specificity at 95%, 38 subjects (18.4%) were selected in the high-risk group, 108 subjects (51.5%) in the low-risk group and the remaining 63 (30.1%) in the intermediate-risk group. Of those classified as high risk 7 (3.3% of the total sample) were actually amyloid negative and therefore classified as FP. Of those classified as low risk, 3 subjects (1.4% of the total sample) were actually amyloid positive in the CSF and therefore considered as FN (Figure 4B). The PPV and NPV were 0.82 and 0.97, respectively; and the global accuracy 0.93.

Setting sensitivity and specificity at 97.5%, 19 subjects (9.1%) were classified as high risk, 22 (10.5%) as low risk and 168 (80.4%) were indeterminate. Of those classified as high risk, 4 (1.2% of the total sample) were FP and there was 1 subject (0.48% of the total) who was FN (Figure 4C). In this case, the PPV, NPV, and global accuracy were 0.79, 0.99, and 0.87, respectively.

## Discussion

In recent months, plasma p-tau217 is emerging as the most promising single biomarker for the diagnosis of AD pathology. It has shown an excellent ability to detect amyloid and tau pathology in cohorts including both healthy and symptomatic subjects, with AUCs between 0.92–0.96 and 0.93–0.97 respectively (35). Our results showed also an excellent diagnostic performance of p-tau217 alone with an AUC 0.85 for detecting amyloid pathology. This is of particular importance considering that all our participants are CU and with a high proportion of A−T−N− subjects (66.9%), so we could consider our population to be particularly demanding. This movement to earlier stages of the AD continuum complements previous work and yields data on the ability of plasma p-tau217 as a potential screening tool. In this regard, our results are also interesting as we have used the Lumipulse G600II platform, which is a fully automated device and widely available in hospitals throughout the world. Recent work have explored the ability of plasma p-tau217 measured with this platform to differentiate between A−/+ subjects and have also found excellent AUCs (close to 0.90) proposing an optimal cutoff point of 0.158 (36). The differences with our optimal cutoff point of 0.095 for p-tau217 alone are probably due to differences in the cohorts, as that work includes patients with cognitive impairment and higher levels of pathology.

When we directly compared plasma p-tau217 with p-tau181, we found that the former detected better CSF amyloid (AUC 0.85 vs 0.75). Moreover, the results of p-tau217 were not significantly different from those of the amyloid ratio. This finding points to a cost-effectiveness advantage since similar results can be obtained by measuring only one biomarker versus the two that we would need to measure using the Aβ42/Aβ40 plasma ratio. In this sense, our work complements information from previous studies in which plasma p-tau217 performed better than other tau species such as p-tau181 and p-tau231 in cohorts including patients with mild cognitive impairment and dementia (37). Taken together, these data suggest that the biomarker is the more reliable in both preclinical and symptomatic stages for detecting amyloid pathology.

The highest correlation of plasma p-tau217 with core biomarkers in CSF was found with p-tau181, with a positive correlation of 0.6. However, since the relationship between plasma p-tau217 and CSF amyloid ratio is not linear, they showed a significant but lower correlation (Rho=−0.51). This is consistent with previous work in which weak correlations have been evidenced between the CSF amyloid ratio and the rest of plasma biomarkers (including the plasma ratio) (38). Our results reflect that, although plasma p-tau217 is a specific biomarker of both amyloid and tau deposition (39), plasma amyloid levels are influenced by other factors such as peripheral expression and cardiovascular drug use as sacubitril-valsartan (40, 41).

On the other hand, similar to former studies, we have seen that p-tau217 levels are significantly higher in A+ subjects (compared to A−) and also in AD+ subjects (compared to AD−) (8). When analyzing the ATN groups, we also found a progressive increase of p-tau217 values across the AD continuum. This suggests that, in addition to being an early biomarker, it continues to increase throughout the different biological phases of the disease and that it could be a useful tool to monitor evolution and response to treatments. Moreover, previous works with longitudinal follow-up have shown that plasma p-tau217 increases over time in A+ subjects with respect to controls and that these changes correlate significantly with cognitive decline and brain atrophy (42, 43).

We have also considered different physiological variables and comorbidities that have been reported to have the greatest influence on plasma biomarkers such as the Aβ42/Aβ40 ratio, p-tau181 and p-tau217 (23, 44–47). In this case, we found no data suggesting that eGFR, BMI or previous diagnoses of HT and DM influence plasma p-tau217 levels. This would add robustness to the viability of the biomarker as a population screening tool since we did find an influence of HT and DM on the values of p-tau181 and Aβ42/Aβ40 (23). However, the findings should be taken with caution because in another study with a larger sample size and higher proportion of kidney dysfunction, subjects with decreased eGFR and HT did have higher plasma p-tau217 levels (46). To avoid the influence of these factors on diagnostic accuracy, it has been proposed the use of the plasma ratio of p-tau217 to non-phosphorylated levels of this protein, which seems to be less influenced by renal function (48).

When we have made the proof of concept of practically applying this biomarker in our population, we have observed that depending on the different cut-off points, between 19.9–81.1% of subjects were outside of the “indeterminated zone” to detect amyloid pathology, thus being candidates to avoid LPs. These numbers are lower than previously reported (21). This finding should not surprise us and does not undermine the usefulness of p-tau217, since our population is composed of cognitively healthy individuals and the proportion of underlying pathology is lower than expected in patients with mild cognitive impairment, thus making it a more challenging population.

Interestingly, recent recommendations from the Global CEO Initiative on AD suggest that amyloid plasma biomarkers should be used as a confirmatory tool only when they have a sensitivity and specificity of at least 90% compared to amyloid PET (49). Biomarkers with at least 90% sensitivity and lower specificities should be used only as a screening tool. These numbers are very close to those described in this paper and complement the idea of a two-step workflow. The model we have selected with p-tau217 and ApoE4 status presents a sensitivity and specificity of 0.93 and 0.73 respectively, very similar to what is required to be used as a screening tool. This findings, in a demanding population as ours, suggests that p-tau217 measured with the Lumipulse platform may be a suitable marker to use as a population screening followed by confirmatory testing when necessary.

Although our findings are sound, internally consistent and in line with those published to date, several limitations should be noted. On the one hand, the sample size may pose a problem of statistical power in some sub-analysis. Although the main findings are quite significant, when stratified analyses are performed, it is likely that some of the comparisons are no longer significant (specifically with the A+T−N− group with a size of 19 subjects). Another limitation is the absence of longitudinal follow-up. In this sense, sequential plasma samples could help to define the different p-tau217 trajectories between A− and + subjects. Moreover, despite having followed international recommendations for plasma collection and storage, it seems that each biomarker may be influenced differently by various factors. The performance of p-tau217, for example, is influenced by centrifugation, but not by the thawing temperature or the number of freeze-thaw cycles (50). Standardization of the diverse analytical steps can be key to minimizing measurement variability.

In conclusion, plasma p-tau217 measured with the fully automated platform Lumipulse, shows a remarkable performance for detecting AD pathological changes in the CSF of cognitively healthy subjects. These data in a preclinical population suggest that p-tau217 plasma may be a good candidate for population screening and subject selection for clinical trials.

## SUPPLEMENTARY MATERIAL

Supplementary material, approximately 100 KB.

## Notes

*Ethics approval and consent to participate:* This research has been conducted in accordance with the Declaration of Helsinki and has been approved by the ethics committee of the Hospital Universitario Marqués de Valdecilla. Title: Valdecilla Cohort for the study of memory and brain aging. Internal code: 2018.111. All subjects have given their signed consent to participate.
