# Supplementary material for: Diagnostic Accuracy of Plasma p-tau217 for Detecting Pathological Cerebrospinal Fluid Changes in Cognitively Unimpaired Subjects Using the Lumipulse Platform
Source: J Prev Alzheimers Dis. 2024 Aug 7;11(6):1581–91. doi: 10.14283/jpad.2024.152 (PMC11573816; doi:10.14283/jpad.2024.152)
Supplement: Supplementary file 1 — Supplementary material, approximately 100 KB. [file 42414_2024_152_MOESM1_ESM.docx]

**SUPPLEMENTARY MATERIAL**

**Contents:**

- **Supplementary Figure 1:** Graphical flowchart for participants selection.
- **Supplementary Table 1:** Correlations between plasma and CSF biomarkers.
- **Supplementary Table 2:** Variable selection process for the logistic regression model taking CSF amyloid status as response.

**Supplementary Figure 1.** Flowchart for participant selection.


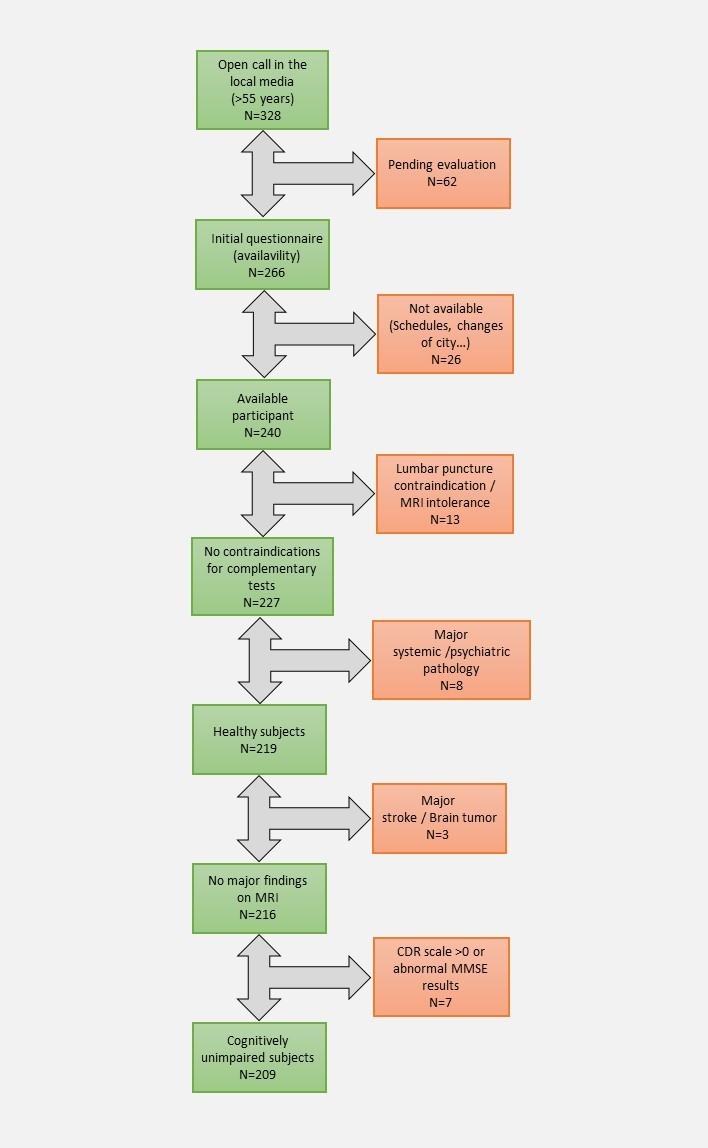


Supplementary Figure 1. Abbreviations: N, number of participants. MRI, magnetic resonance imaging. CDR, clinical dementia rating. MMSE, mini-mental state examination.

**Supplementary Table 1.** Correlation between plasma and CSF biomarkers.

|  | | | PLASMA BIOMARKERS | | | | | |
| --- | --- | --- | --- | --- | --- | --- | --- | --- |
|  |  |  | **Aβ42/Aβ40 ratio** | | **P-tau181** | | **P-tau217** | |
|  |  |  | r | p-value | r | p-value | r | p-value |
| CSF BIOMARKERS | **Aβ40** | Overall | -0.23 | **<0.001** | 0.11 | 0.11 | 0.07 | 0.37 |
|  |  | A- | -0.21 | **0.01** | 0.07 | 0.30 | 0.05 | 0.51 |
|  |  | A+ | -0.13 | 0.35 | 0.05 | 0.72 | 0.04 | 0.75 |
|  | **Aβ42** | Overall | 0.27 | **<0.001** | -0.20 | **0.003** | -0.33 | **<0.001** |
|  |  | A- | -0.12 | 0.13 | 0.07 | 0.42 | -0.07 | 0.36 |
|  |  | A+ | 0.18 | 0.16 | -0.17 | 0.22 | -0.24 | 0.06 |
|  | **Aβ42/Aβ40 ratio** | Overall | 0.6 | **<0.001** | -0.38 | **<0.001** | -0.49 | **<0.001** |
|  |  | A- | 0.18 | **0.03** | -0.01 | 0.88 | -0.05 | 0.54 |
|  |  | A+ | 0.34 | **0.009** | -0.24 | 0.06 | -0.34 | **0.007** |
|  | **P-tau181** | Overall | -0.47 | **<0.001** | 0.47 | **<0.001** | 0.59 | **<0.001** |
|  |  | A- | -0.21 | **0.01** | 0.06 | 0.49 | 0.02 | 0.78 |
|  |  | A+ | -0.28 | **0.03** | 0.46 | **<0.001** | 0.61 | **<0.001** |

Supplementary Table 1: Correlations between plasma and CSF biomarkers using Pearson's correlation coefficient. Significant results are highlighted in bold. Abbreviations: Aβ, amyloid beta. P-tau, phosphorylated tau. A, amyloid status. r, Pearson’s correlation coefficient. P-value, statistical significance.

**Supplementary table 2.** Variable selection process for the logistic regression model taking CSF amyloid status as response.

|  | Predictor coefficients,  p-value | | | Fit metrics | | | Likelihood ratio test vs full model | | Frequency of selection at backward variable selection |
| --- | --- | --- | --- | --- | --- | --- | --- | --- | --- |
| Model | **P-tau217** | **Age** | ***ApoE*4 status** | **R^2^** | **AIC** | **AUC** | **X^2^** | **P-value** |  |
| P-tau217 +  Age +  *ApoE*4 status | 9.4, p=0.004 | 0.04, p=0.16 | 1.91, p<0.001 | 0.30 | 176.6 | 0.87 | - | - | 16.7% |
| P-tau217 +  *ApoE*4 status | 10.38, p<0.001 | - | 1.84, p<0.001 | 0.30 | 176.5 | 0.88 | 1.87 | 0.17 | 82.9% |
| P-tau217 | 11.1, p<0.001 | - | - | 0.19 | 196.8 | 0.85 | 25.5 | <0.001 | 0.4% |

Supplementary Table 2: Similar to previous work (1,2) we started from a full logistic regression model to detect CSF amyloid positivity. This model included plasma p-tau217 values, age and ApoE4 status. A backward selection of variables was performed by bootstrapping (n=1000). The first column shows the different models identified and the last column shows the frequency with which each model was selected in the bootstrapping process. The second column shows the regression coefficients of each variable for each model and its p-value. The fourth column shows quality of fit metrics such as the coefficient of determination (R2), the Akaike's information criterion (AIC); and the area under the curve (AUC). The fifth column shows the chi-square value (X2) and the p-value of the likelihood ratio tests between the full model and each of the candidates. In each bootstrap procedure, model variables were removed from the full model if they did not meet p=0.157 in the likelihood ratio tests. Abbreviations: P-tau, phosphorylated tau. ApoE, apolipoprotein E. P-value, statistical significance. R^2^, coefficient of determination. AIC, Akaike’s information criterion. AUC, area under the curve. X^2^, chi-square.
